# Profil des insuffisants rénaux chroniques diabétiques à l'initiation de l'hémodialyse au service de néphrologie et dialyse de l'hôpital militaire de Rabat, Maroc

**DOI:** 10.11604/pamj.2013.15.124.2252

**Published:** 2013-08-07

**Authors:** Mohamed Reda El Farouki, Abdelaali Bahadi, Mohamed Amine Hamzi, Driss Kabbaj, Mohammed Benyahia

**Affiliations:** 1 Service de néphrologie et dialyse de l'hôpital militaire d'instruction Mohammed V, Rabat, Maroc

**Keywords:** Diabète, insuffisance rénale chronique, hémodialyse, Diabetes, chronic renal failure, hemodialysis

## Abstract

Le diabète constitue une cause fréquente d'insuffisance rénale chronique terminale (IRCT) dans le monde. Ce travail présente une étude clinique rétrospective dont le but est de décrire le profil clinico-biologique des patients diabétiques en IRCT, de le comparer aux patients non-diabétiques au stade d'IRCT, et de suivre l’évolution de leurs abords vasculaires, afin d'en déduire des conclusions sur une prise en charge particulière des patients diabétiques. Les paramètres cliniques et biologiques concernant les patients mis en hémodialyse dans notre formation entre le 01 janvier 2006 et le 31 décembre 2011, ont été recueilli et analysés. Nous avons procédé à l’étude comparative des patients en fonction de l'existence ou non d'une néphropathie diabétique, et nous nous sommes intéressés à l’évolution de leurs abords vasculaires. Il s'agit de 207 patients insuffisants rénaux chroniques, dont 86 diabétiques. Le groupe des patients diabétiques était moins suivi avant la mise en hémodialyse (3,66 mois vs. 6,32 mois), avec une prise beaucoup plus importante d'antihypertenseurs (1,87 vs. 1,14, p<0,001). L’échec des abords vasculaires était plus important chez les patients diabétiques (45% vs. 27%, p=0,006), avec une survie moyenne plus faible de leurs abords vasculaires (509 vs 753 jours, p=0,003). L’étude comparative des taux d'hémoglobine, de parathormone intacte, d'albuminémie et de C-réactive protéine, entre le groupe de patients diabétiques et non diabétiques était non significative. Notre étude soulève le problème du suivi néphrologique chez les diabétiques, pourtant censés être mieux suivis, et son retentissement sur l'avenir de leurs abords vasculaires.

## Introduction

Le diabète constitue la première cause d'insuffisance rénale chronique (IRC) dans les pays industrialisés [1]. Aux stades précoces, des traitements néphroprotecteurs associés à l’équilibre glycémique et à la modification de l'hygiène de vie sont recommandés, mais au stade terminal, la survie est assurée par un traitement de suppléance: dialyse ou transplantation rénale. Alors que le nombre de personnes diabétiques de type 1 traités pour insuffisance rénale chronique terminale (IRCT) semble se stabiliser, voire diminuer, dans le monde, le nombre de personnes diabétiques de type 2 en IRCT ne cesse d'augmenter [2, 3]. L'incidence de la néphropathie diabétique au stade terminal est variable selon les pays: 44% aux Etats-Unis en 2008 [4], 34% en Australie-Nouvelle-Zélande en 2008 [5], 11,8% à 35,5% selon les pays en Europe en 2007 [6], et 22,9% en France en 2008 [7].

Ce travail présente une étude clinique rétrospective dont le but est de décrire le profil clinico-biologique des patients diabétiques en IRCT, de le comparer aux patients non-diabétiques au stade d'IRCT, et de suivre l’évolution de leurs abords vasculaires, afin d'en déduire des conclusions sur une prise en charge particulière des patients diabétiques.

## Méthodes

Les données concernant les patients mis en hémodialyse, entre le 01 janvier 2006 et le 31 décembre 2011 au sein du service de néphrologie, dialyse et transplantation rénale de l'hôpital militaire d'instruction Mohammed V, ont été rétrospectivement recueillies. Nous nous sommes intéressés aux caractères cliniques, et aux données biologiques (taux d'hémoglobine, cholestérol total, LDL, HDL, triglycérides, albuminémie, ferritinémie, C-Reactive protein) au moment de la mise en hémodialyse. Nous avons procédé à l’étude comparative des patients en fonction de l'existence ou non d'une néphropathie diabétique, et nous avons suivi l’évolution de leurs abords vasculaires.

La collecte des données a consisté en un recueil des caractéristiques démographiques, la présence ou non d'une néphropathie diabétique, l'existence de comorbidités, la prise et le nombre d'antihypertenseurs, le suivi néphrologique avant la mise en hémodialyse, ainsi que sa durée.

L’évaluation des abords vasculaires a été effectuée le 31 décembre 2011. Leur survie a été calculée depuis le jour de la confection jusqu'au jour de l’échec, ou jusqu'au 31 décembre 2011. L’échec primaire a été défini comme l’échec de l'accès vasculaire avant l'initiation de l'hémodialyse ou l'absence de maturation dans les six mois suivant sa création.

L'analyse statistique a été réalisée par le logiciel SPSS version 11.Nous avons utilisé le test de Student pour les variables quantitatives et le test khi^2^ pour les variables qualitatives. Le seuil de significativité est considéré positif si le p est inférieur à 0,05.

## Résultats

**Figure 1:**
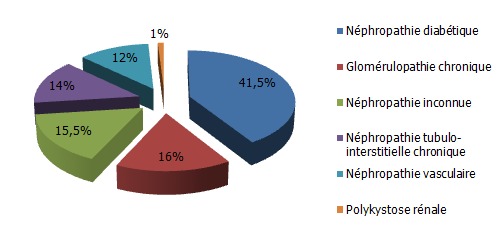
Néphropathie causale des patients mis en hémodialyse

Il s'agit de 207 patients insuffisants rénaux chroniques, dont 86 diabétiques (41,5%) (Figure 1). L’âge moyen des patients est de 52 ans, le sex-ratio est de 0,55. (Tableau 1)

**Tableau 1:** Caractéristiques démographiques et clinico-biologiques des patients

Facteur	Valeurs
Nombre de cas	207
Age (année)	52,43 ± 15,48
Sexe masculin (N/%)	133 / 64,3
Suivi néphrologique avant mise en hémodialyse (N/%)	67 / 32,4
Suivi néphrologique moyen (mois)	5,2 (0- 48)
Hypertension artérielle (N/%)	153/ 73,9
Diabète (N/%)	86 / 41,5
Hémoglobine (g/dl)	8,86 ± 2,01
Albuminémie (g/l)	31,17 ± 7,02

Le groupe des patients diabétiques était moins suivi avant la mise en hémodialyse, (3,66 mois vs. 6,32 mois, p=0,07), avec une prise beaucoup plus importante d'antihypertenseurs (1,87 vs. 1,14, p<0,001). 63% des diabétiques ont bénéficié de leurs premières séances d'hémodialyse via des cathéters veineux temporaires, contre 58% dans le groupe des patients non-diabétiques.

L’étude comparative des taux d'hémoglobine, de parathormone intacte, d'albuminémie et de C-réactive protéine, entre le groupe de patients diabétiques et non diabétiques était non significative, avec des résultats successifs (8,9 g/dl vs. 8,7 g/dl, p=0,42), (444,5 pg/ml vs. 516 pg/ml, p=0,22), (29,9 g/l vs. 31 g/l, p= 0,06), et (24,9 mg/l vs. 23,7 mg/l, p=0,86), le taux de ferritinémie était par contre significativement plus bas chez les diabétiques (267 ng/ml vs. 343 ng/ml, p= 0,04).

L’échec des abords vasculaires était plus important chez les patients diabétiques (45% vs. 27%, p=0,006), avec une survie moyenne plus faible de leurs abords vasculaires (509 vs 753 jours, p=0,003). La durée d'hospitalisation était plus courte chez les diabétiques que les non-diabétiques (10 vs. 13,1 jours, p=0,01). (Tableau 1)

## Discussion

A l'heure actuelle, environ le tiers des patients en IRCT débutant l'hémodialyse périodique sont diabétiques [7]. L'incidence des patients diabétiques en IRCT a atteint 35% en France en 2006 de l'ensemble des patients en IRCT [7]. Sur le registre ANZDATA (ANZDATA, Australia and New Zealand Dialysis and Transplant Registry; 1991- 2005), l'incidence annuelle moyenne de l'IRCT due au diabète de type 1est de 5 par million d'habitants (pmh), contre 10,6 pmh en 1991 et 48,8 pmh en 2005 pour le diabète de type 2 [5]. Aux Etats-Unis, la prévalence des diabétiques en IRCT a atteint 37% de l'ensemble des IRC terminaux (2008), avec une incidence annuelle à 44% (2007) [4]. Dans notre étude, la prévalence des diabétiques (41,5%) rejoint celle des séries sus-décrites.

Les diabétiques de notre série étaient significativement plus âgés que les non-diabétiques à l'initiation de l'hémodialyse (59,7 ans vs. 47,1 ans, p<0,001). En Côte d'Ivoire, Roux Amani a rapporté un âge moyen de 53 ± 6,7 ans dans une série de 759 diabétiques [8]. L’âge relativement élevé des malades de notre série peut être expliqué par la référence tardive des malades en néphrologie, et l'initiation tardive de l'hémodialyse au stade de complications le plus souvent. La référence tardive chez le néphrologue, évaluée dans notre série par le suivi avant la mise en hémodialyse a concerné la majorité des patients de notre série, puisque seulement 32% de nos patients avaient un suivi spécialisé d'au minimum 4 mois avant leur mise en hémodialyse; paradoxalement les diabétiques ‘ porteurs d'une maladie chronique ‘ et par conséquent, censés être mieux suivis, sont les moins suivis, (3,66 mois vs. 6,32 mois, p=0,07). Cette fréquence de référence tardive est comparable à celles des autres séries européennes ou américaines publiées après 1995 [9–12]. Elle témoigne d'une situation fréquente et internationalement constatée. Les études réalisées au sein des mêmes équipes et à des périodes différentes depuis 1984, témoignent en outre de la relative constance du phénomène [13, 14].

Le suivi spécialisé influence la qualité de la prise en charge au moment de la mise en hémodialyse. Ainsi, le taux d'hémoglobine moyen est de 8,86 ± 2,01 g/dl, sans aucune différence significative entre les deux groupes de patients: diabétiques et non diabétiques (8,9 g/dl vs. 8,7 g/dl, p=0,42). Sur le registre REIN en 2006, 2 patients sur 3 en IRCT avaient un taux d'hémoglobine inférieur à 11 g/dl à l'initiation du traitement, en dépit de l'accessibilité à l’érythropoïétine, qu'ils soient ou non diabétiques [8, 15]. De même que dans une étude précédente [16], en France, il n'a pas été mis en évidence de lien entre le statut diabétique et l'importance de l'anémie, contrairement à ce qui a pu être observé dans l’étude ACORD (The Anaemia CORrection in Diabetes) [17]. Le taux de ferritinémie significativement plus bas chez les diabétiques de notre série pourrait y contribuer de sa part (267 ng/ml vs. 343 ng/ml, p= 0,04).

Le diabète apparait comme un facteur altérant la survie des fistules artério-veineuses. La médiacalcose, la dysfonction endothéliale, ainsi que l'augmentation du stress oxydatif responsable d'une augmentation des événements thrombotiques, contribuent à l’échec des abords vasculaires chez les diabétiques [18–20]. L'exploration systématique du membre supérieur avant la confection de l'abord vasculaire diminuerait de façon significative le risque d’échec de 23% selon Branger [21].

## Conclusion

Le diabète, de par sa chronicité et la systématisation de son suivi continue de constituer une cause fréquente d'IRCT. L'absence de suivi néphrologique chez les diabétiques, accentue les morbidités associées au moment de la mise en hémodialyse, et altère la survie de leurs abords vasculaires. Un transfert précoce chez le néphrologue dès la néphropathie diabétique débutante s'impose, afin d'optimiser la prise en charge des complications de l'IRC due au diabète, et d'améliorer la survie de leurs fistules artério-veineuses.
